# Perspective, Opportunities and Challenges in Using Fennel (*Foeniculum vulgare*) in Poultry Health and Production as an Eco-Friendly Alternative to Antibiotics: A Review

**DOI:** 10.3390/antibiotics11020278

**Published:** 2022-02-20

**Authors:** Rifat Ullah Khan, Adia Fatima, Shabana Naz, Marco Ragni, Simona Tarricone, Vincenzo Tufarelli

**Affiliations:** 1 Faculty of Animal Husbandry and Veterinary Sciences, College of Veterinary Sciences, The University of Agriculture, Peshawar 25130, Pakistan; 2 Department of Poultry Science, Faculty of Animal Husbandry & Veterinary Sciences, The University of Agriculture, Peshawar 25000, Pakistan; adiis3576@gmail.com; 3 Department of Zoology, Government College University, Faisalabad 38000, Pakistan; drahabananaz@gxfu.edu.pk; 4 Department of Agro-Environmental and Territorial Sciences, University of Bari ‘Aldo Moro’, 70126 Bari, Italy; marco.ragni@uniba.it (M.R.); simona.tarricone@uniba.it (S.T.); 5 Department of DETO, Section of Veterinary Science and Animal Production, University of Bari ‘Aldo Moro’, 70010 Bari, Italy

**Keywords:** fennel, health, production, poultry, antibiotics

## Abstract

Following the European Union’s restriction on antibiotic growth promoters, research on enhancing gut health has been accelerated. As the poultry industry is facing issues that were previously managed by antimicrobial growth promoters, the hunt for the best remedies continues to find suitable alternatives. Simultaneously, social pressure is mounting to reduce the usage of antibiotics and replace them with other feed additives. Consumers believe a number of accessible options to be safe, with phytogenics playing a crucial role. This review describes how the use of fennel seeds could be beneficial for poultry. An overview of the broad chemical diversity of fennel is presented together with their physicochemical and biological properties. According to investigations, fennel seeds have a variety of biological effects in birds, including improved performance, higher immune cell proliferation, reduced oxidative stress, and boosted antibody titers against infectious diseases. The efficacy of poultry outcomes is determined by the stage and age of the plants, the extraction process, the geographical location, the chicken species, management techniques, and the concentrations administered. The present review focuses on the effects of fennel seeds as a feed additive on poultry growth, carcass quality, blood biochemistry, antioxidant activity, immunity, and microbiological aspects.

## 1. Introduction

**Figure 1:**
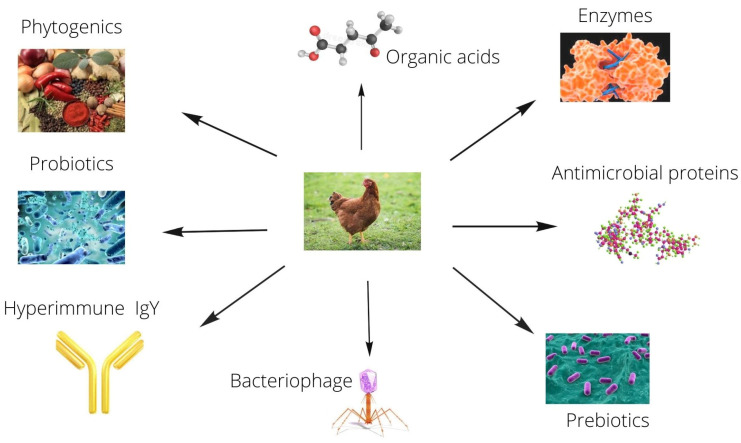
Different feed additives reported in poultry production.

Dietary antibiotics have undoubtedly played an important role in animal production as a growth and health booster. However, due to public concerns about antibiotic use in feed and the accompanying residues, as well as the emergence of antibiotic-resistant bacteria, there is a current tendency to explore for alternatives to antibiotics. Dietary antibiotics are thought to increase animal performance by modulating intestinal microbiota. The majority of products marketed as antibiotic alternatives have direct or indirect impacts on the gut microbiota. Important alternatives to antibiotics reported in poultry are probiotics [1,2,3,4,5,6,7,8] prebiotics [9,10,11,12], enzymes [13,14,15,16,17], antimicrobial peptides [18,19], bacteriophage [20,21] hyperimmune IgY [22,23], organic acids [24,25,26], and phytogenics [27,28,29,30,31,32,33,34,35,36,37,38,39], as shown in Figure 1.

Phytogenics have been described as growth-promoting and alternative to antibiotics in animal production [40,41,42,43] Phytogenics feed additives are natural medicinal items produced from herbs and spices that are used in livestock nutrition to improve performance and health [39,44,45,46,47,48,49] The use of phytogenic feed additives or herbal plants as antibiotic alternatives has lately gained a lot of attention. Because these phytogenic plants are natural products, people may be prepared to accept their use in poultry diets. Phytogenic plants have been shown to have growth-promoting, antibacterial, antioxidant, and anti-inflammatory properties, according to extensive research [50,51] Furthermore, they stimulate the digestive system by raising the generation of digestive enzymes and improving feed utilization efficiency through improving liver activities. However, our understanding of how they may be used in poultry nutrition is currently little attended.

**Figure 2:**
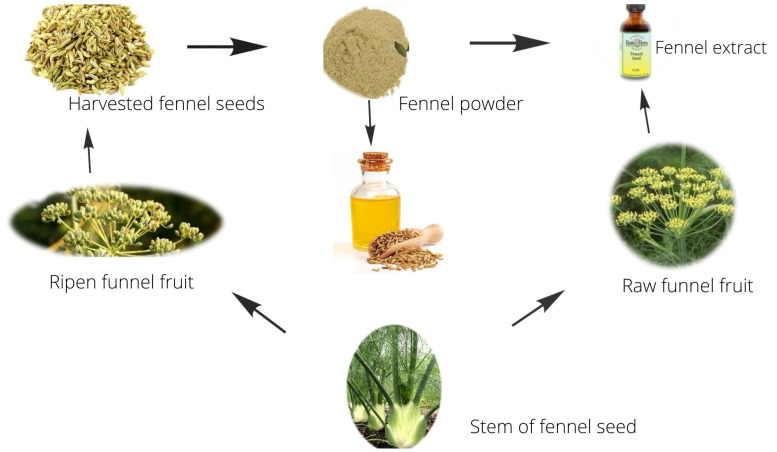
Different forms of fennel plants and seeds.

Fennel (*Foeniculum vulgare*) is a perennial herb native to southern Europe and the Mediterranean region that grows upright. The plant has yellow blooms on a complex umbel and grows to about 1.5 m tall. Different forms of fennel plant and seeds are given in Figure 2. 

The oil content ranges from 0.6 to 6%; fruits closer to the center of the umbel are often larger, greener, and hve a stronger perfume. Some basic information and chemical composition of fennel seeds is given in Table 1 and Table 2. 

**Figure 3:**
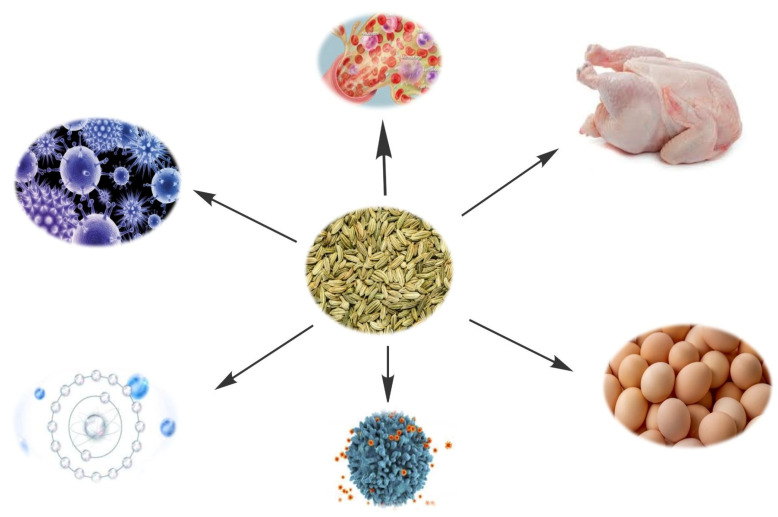
Multiple beneficial effects of fennel seeds in poultry.

In the food and flavour industry, fennel seeds areused in meat, vegetable products, fish sauces, soups, salad dressings, stews, breads, pastries, teas, and alcoholic beverages. Fennel oil is used in sauces, soaps, creams, perfumes, and liqueurs, among other things. For aesthetic purposes and as a raw vegetable, fennel comes in a range of morphologies and leaf colors. As a medicinal plant, fennel seed has been used as a carminative, antispasmodic, expectorant, diuretic, stimulant, laxative, and stomachic [54]. In the essential oil of fennel seeds, one of the most active component, 1-methoxy-4-(E)-propenyl-benzene has been discovered [55] which has multiple biological functions. According to considerable research on the plant’s leaves and fruits, fennel essential oil has outstanding antioxidant, antimicrobial, and hepatoprotective properties [56]. Fennel is one of the herbs that include a significant percentage of the fatty acids linolenic acid and stearic acid, according to [57]. Furthermore, fennel essential oil includes 16.81% trans-anethol and 47.20% estragole and totaling 64.01% suger. According to [58] fennel is known for fennel extensive antioxidants and biological properties. Fennel seed has been used as a laxative, expectorant, spasmolytic, anti-colic, and digestive enzyme stimulant for thousands of years [59]. Fennel essential oil contains many compounds, including limonene, fenchone, phellandrene, cisocimene, para-cymene, gamma-terpinene, anethole, alpha-pinene, camphene, sabeinene, beta-myrcene, estragole, safrole, beta-pinene, camphor and other volatile components as well as affixed oil [60]. In view of the above mentioned properties of fennel seeds, this review was compiled on the growth performance, antioxidant, immunological, antimicrobial activity as well as serum biochemistry of poultry. Some of the detail of the previous research work has also been presented in Table 3 and illustrated in Figure 3.

## 2. Uses of Fennel Seeds as an Alternative to Antibiotics

### 2.1. Growth Performance in Poultry 

Recently, Saleh et al. [76] and Al-Sagon et al. [61] found that supplementing broiler diets with fennel seed powder boosted feed intake under heat stress conditions. In a similar study, Saki et al. [65] found that feeding fennel seed to broilers enhanced feed consumption. Ragab [67] and Henda et al. [66] found that adding fennel seed to the diet boosted feed consumption in Japanese quails. However, Bugdayci et al. [74] found that adding fennel seed to the diet had no effect on feed consumption. The increase in feed intake can be linked to the improved palatability of the feed as well as the fennel’s odour. Natural feed additives have beneficial effects for stimulating and activating the digestive system by improving the palatability of the diet and increasing the appetite of poultry, resulting in increased feed consumption. Furthermore, the antibacterial and antifungal effects aid in better digestion of nutrients, resulting in increased feed consumption [88] However, Soltan et al. [71] Abou-Elkhair et al. [67] and Zahira Abul-Jabbar et al. [69] found that adding fennel powder to the broiler diet reduced feed consumption. Gharghani et al. [70] on the other hand, found that including fennel seeds in the diet had no effect on the amount of feed consumed by layers. Similarly, Ali Safaei et al. [81] found that adding fennel seed to the diet of broilers had no effect on feed consumption. These contradictory results could be owing to the concentration of fennel active components and their level in the meal.

The presence of essential oil and active ingredients in fennel seed such as anethole and estragol, which stimulate the secretion of bile acid and digestive enzymes like protease, lipase, amylase, and maltase, facilitate digestion, may be the reason for increased feed consumption in fennel supplemented birds [89] Fennel seed has been shown to increase hunger, boost endogenous digestive enzymes, and trigger immunological response [66]. Fennel, like other medicinal plants, has antibacterial and antibiotic properties that may help to reduce the quantity of unwanted intestinal microorganisms and improve digestion [90]. Some authors have attributed the enhanced feed consumption to the trans-anethole, estragole, anethole and oestragole of fennel seeds [57,91].

Improved weight gain and feed conversion ratio in response to fennel seeds supplementation has been reported in broilers, layers and Japanese quails in different doses and preparations [61,65,67,68,69,70,76,79,80,81,92,93,94]. Some researchers believe that the presence of fat soluble unidentified factor (a mixture of important fatty acids including linoleic, linolenic, and arachidonic acids) and vitamins in fennel seeds contributes to improved body weight gain [95]. According to [96] fennel seeds stimulate the flow of digestive juices, convert fats to fatty acids, reduce disease-causing microbes in the digestive tract, and raise live body weight [96]. Improved performance and carcass quality in chicken treated with fennel seed powder and oils may be attributed to improved digestibility and an expanded antioxidant profile, according to researchers [61,78]. Fennel also possesses potent antiviral, antibacterial, and anti-inflammatory properties that may help to enhance gut health and remove infections. Essential oils (methyl chavicol, limonene, anethole, fenchone, phellandrene, anisic acid, camphene, palmitic, oleic, linoleic, pinine, and petroselenic acids, volatile chemicals, and flavonoids) are abundant in fennel seeds powder [52,70] and may cause possible growth improvement in poultry.

### 2.2. Egg Production and Quality Traits

Kazemi et al. [82] reported that hens fed 50 mg/kg fennel extract had significantly higher egg production and increased shell thickness in laying hens. On the other hand, Bozkurt et al. [86] observed that egg production was not affected by essential oil premixes containing fennel throughout 22 to 45 weeks of laying periods. Similarly, Nasiroleslami et al. [80] reported that the effect of adding fennel essential oil on egg quality traits were not statistically significant, except for Haugh unit as well as egg shell quality. In a study, phytoestrogen supplementation increased performance and improved egg quality variables in quails [97] and therefore, it is suggested that fennel has oestrogen-like compounds which induce egg production. Dietary fennel supplementation in the diets of laying hens has been found to reduce the deleterious effects of heat stress on egg quality indices [70].

Fennel seed essential oil has been demonstrated to have appetite-stimulating, digestive enzyme and bile acid secretion-enhancing, and antioxidant properties [68]. probably due to the presence of protein, carbohydrate, minerals and vitamins. It also is likely that these phytogenic feed additions increased egg yolk precursor hepatic production by shielding hepatocytes from oxidative damage, resulting in improved yolk formation and ovulation [98]. Gharaghani et al. [70] and Vakili and Majidzadeh Heravi [99] both found that adding fennel to laying hen diets boosted egg weight in both heat stress and normal ambient temperatures. The favourable impacts of phytogenic feed additives in altering gut microbiota, boosting food digestibility and absorption, and improving ovarian characteristics resulted in improved health status and subsequent laying performance [10,100].

Fennel addition in laying hen diets under heat stress raised Haugh unit, which is a measure of egg quality inside the shell, according to Gharaghani et al. [70] The antibacterial and antioxidant properties of phytogenics might explain the increased albumin weight. Herbal plants’ bioactive compounds have also been demonstrated to preserve the magnum and uterus, as well as increase albumen production in laying birds. Nasiroleslami [80] demonstrated that dietary fennel essential oil lowered Haugh unit and enhanced eggshell weight and thickness in laying hens. In addition to egg production and egg mass, yolk color was improved in laying hens in the study of Abou-elkhair et al. [68]. The presence of carotenoids pigment might explain the higher egg yolk colour score in the fennel supplemented groups. In the laying quails, egg quality parameters were not affected by supplementation of different levels of fennel seeds as reported by [74]. In broiler breeders, egg production, Haugh unit and eggshell thickness were improved in response to fennel supplementation [82]. Recently, Souza et al. [101] reported that egg and albumin weight were improved in laying quails fed with 750 mg fennel. In 55-week-old white shaver hens, egg quality parameters such as egg production, egg weight, yolk weight were significantly influenced in response to 10 mg oral dose of fennel extract. 

Following the application of medicinal plants in poultry feeding, a larger amount of calcium is deposited on the eggshell, which is due to an increase in the secretion of various digestive enzymes as well as an improvement in the intestines’ anatomical status for the uptake of various nutrients, including calcium. Fennel is thought to enhance the size of oviducts, causing them to become more active in terms of producing albumin proteins, shell membrane, and the calcium carbonate essential for shell production [83]. Yazarlou et al. [102] concluded that fennel seed levels have a substantial impact on the relative weight of the ovary, egg duct, and oviduct. These effects might possibly enhance egg production and quality. 

### 2.3. Antimicrobial and Immune Stimulating Effects

The antibacterial activity of the essential oil and extracts from fennel seeds was tested against a panel of food-borne and pathogenic microorganisms by Anwar et al. [53]. They found that fennel essential oils had extensive antibacterial activity against all of the bacteria they examined, especially Gram-positive bacteria. The findings of the disc diffusion method revealed that *Bacillus subtilis* and *Aspergillus niger* were the most sensitive bacteria tested, with the greatest inhibition zones. Gram-negative bacteria, particularly *E. coli*, are less sensitive to fennel essential oils, according to Cantore. et al. [103]. Fennel essential oils have been reported to inhibit a wide range of *Bacillius* species, according to [104]. Fennel essential oils are also active against *Aspergillus* species, according to Mimica-Dukić [105]. Ghiasvand et al. [72] reported that essential oil of fennel reduced *E. coli* population in the intestines of broiler chickens. 

According to Gende et al. [59] fennel essential oil exhibits considerable antibacterial action due to its active component, anethole, which was shown to be particularly abundant in the oil of fennel (92.7 percent). Isolated anethole from fennel seeds was compared to conventional anethole, Barrahi et al. [106] found that it was efficient against a variety of microbes, including bacteria, yeast, and fungal strains. The hydrophobicity of essential oils and their components is thought to be a crucial property that allows essential oils to accumulate in the lipid bilayer of the bacterial cell membrane and mitochondria, disrupting cell structures and making them more permeable [107,108]. Furthermore, certain essential oils’ antibacterial function involves disrupting cell homeostasis, which results in growth inhibition and cell death [109]. Nonetheless, it has been suggested that fennel’s chemical structure, such as the presence of a functional hydroxyl (–OH) group and aromaticity, are also responsible for its antibacterial effect [110].

According to Kazemi et al. [83] adding 50 mg/kg of fennel extract to the diet improved the antibody titer against Newcastle disease (ND) in broilers. Soltan et al. [71] investigated the effect of fennel seeds on bird immunity, finding that included fennel seed in the feed of broilers dramatically raised antibody titers against ND. Supplementation of fennel seed oil with a mannan oligosaccharide combination showed no effect on bird immunity, according to [85]. According to Ali-Safaei et al. [81] adding fennel extract to the diet of broilers enhanced Newcastle vaccination efficiency on day 35 and immunoglobulin synthesis on day 42, resulting in improved immunity against bacteria, viral infections, and new infections. in the study of Soltan et al. [71] adding 1.5 g/kg fennel seed to the broiler diet boosted phagocytic activity. At 42 days of age, Bozkurt et al. [86] found that adding essential oil to the diet had no effect on the particular immune response of broiler chickens as determined by serum infectious bursal disease (IBD) and ND viruses.

Relative weight of lymphoid organs such as spleen and thymus are very important for improved immune response. In most of the cases, the weight of lymphoid organs is improved when the immunity level is heightened. Ghiasvand et al. [72] found no significant variations in antibody titres against avian influenza and ND viruses, primary and secondary immunological (total, M, and G immunoglobulin) responses in broilers after dietary supplementation with fennel essential oil. In the same study, adding fennel essential oil to the broiler diet had no effect on blood lymphocyte and heterophile percentages or the heterophil to lymphocyte ratio.

The emergence of better immune response in chickens when fennel seeds are supplied in the diet is poorly understood. Antioxidant qualities and bioactive substances are thought to play a role in the development of the immune response in birds by shielding cells from oxidative damage and improving their function and proliferation [111]. The elevation in serum levels of triiodothyronine and thyroxine caused by fennel seed could explain the differences in immunological response. Triiodothyronine is the first thyroid hormone that stimulates the immune system. Increased thyroid hormone levels are required to provide the energy required for the conversion of bone marrow cells to plasma cells, which results in antibody production [112].

According to Henda et al. [66] adding fennel seed to broiler feed enhanced the relative weight of the spleen and thymus. Fennel extract supplementation in quail meal enhanced relative weight of lymphoid organs, according to Ragab [67] However, fennel seed has no influence on the relative weight of the spleen and bursa in broilers, according to Abdullah and Abbas [79]. The addition of fennel seed to the diet had no influence on the relative weight of lymphoid organs [71]. This fluctuation in lymphoid organ relative weight could be attributed to the active chemicals in fennel seed, as well as thyroid hormones, which stimulate the immune system of birds and provide energy for the conversion of plasma cells to B-cells [113].

### 2.4. Antioxidant Activity

Supplementation with anethol and fenchon (major components of the fennel) has been shown to inhibit the hepatic 3-hydroxy3-methylglutaryl CoA (HMG-CoA) reductase activity, resulting in lower hepatic lipid peroxidation via the enhancement of hepatic antioxidant enzyme activities in broilers [61]. Under normal settings (ambient temperature), Gharaghani et al. [70] found that include fennel fruit in laying hen meals had no discernible influence on performance, egg production, or egg quality. Consumption of fennel fruit as a natural antioxidant may prevent the negative effects of free radicals in laying hens during heat stress circumstances, when the production of oxidative products is high. According to Akbarian [113] supplementation with phenolic compounds from fennel seed dramatically reduced MDA levels in broilers exposed to heat stress throughout the experiment. Treatment of fennel (400 mg/kg) in a mixture with other feed additives reduced lipid peroxidation and restored thiol content and catalase (CAT) activity to normal levels, according to Samadi no-shahar et al. [114]. Fennel extract can raise serum levels of superoxide dismutase (SOD), according to Nahid et al. [115]. This extract, at the above-mentioned concentration, can raise glutathione peroxidase (GPx) levels in comparison to control groups, and fennel extract at these amounts can also raise TAC serum levels in comparison to controls. Recently, Ghiasvand et al. [72] reported that inclusion of fennel oil improved the hepatic antioxidant status of broiler chickens.

The antioxidative effect of fennel seed as a feed additive on the quality of chicken meat was investigated in contrast to a control diet with conventional feed additives (antibiotic and probiotic). Fennel-eating birds had the lowest levels of oxidative compounds in their meat, while antibiotic-eating birds had the highest levels. The findings showed that the chicken meat’s oxidative stability could be affected by the base diet and feed additives, and that fennel as a feed addition may improve the oxidative quality of chicken meat [87]. Free radicals promote peroxidation of membrane lipids, which raises liver enzymes and strengthens the body’s defense system [116]. Phenolic chemicals in fennel seed are particularly important plant elements because of their scavenging capacity due to their hydroxyl groups [117]. The presence of elevated amounts of phenolic compounds and flavonoid in fennel was discovered to have a free radical scavenging activity [52]. Fennel seed powder and ethanolic extract contains a variety of polyphenolic chemicals with antioxidant potential [103,118].

### 2.5. Hematology and Biochemistry

According to Abdullah and Abba [79] s [broilers fed fennel seed at 1, 2 and 3 g/kg showed greater red blood cell count (RBC), haemoglobin (Hb), and packed cell volume (PCV). The H/L ratio of chicks given 2 and 3 g of fennel seed per kg decreased RBC, Hb, and PCV might be due to the improved metabolism and increased nutritional absorption. Except for cholesterol and glucose levels, Raga et al. [67] discovered no significant effects of fennel seed on blood components in quails. In this study, females exhibited substantially greater triglycerides, aspartate aminotransferase (AST), alanine aminotransferase (ALT), total protein, and albumin levels than males in the same trial. In terms of calcium and globulin concentration, there were no significant differences between the sexes. According to Gharehsheikhlou et al. [62] the use of various quantities of fennel and savory essential oils, as well as their combination, in the feed of broiler chickens enhanced the total cholesterol to high density lipoprotein (HDL) ratio. Fennel caused a non-significant rise in blood total protein albumin and globulin in developing quail [66]. According to Ali Safaei et al. [81] adding fennel extract to the feed of broilers had no significant effect on glucose, triglycerides, low-density lipoproteins (LDL), or alkaline phosphatase levels, whereas increasing fennel extract level in the diet boosted HDL levels. According to Abd El-Latif et al. [93] adding fennel to Japanese quail diets increased plasma total protein, albumin, and globulin levels. The presence of phenolic chemicals in fennel, which have negative effects on liver functioning, might explain the rise in blood AST and ALT. In the study of Buğdaycı et al. [74] serum cholesterol did not change significantly in laying quails. Taherkhani et al. [83] reported that by activating hydroxylase, fennel extract produces active D3 vitamin form, which improves calcium absorption in the digestive tracts and hence raises blood calcium levels. Further in the same study, the level of blood estrogen was higher in fennel supplemented laying hens and concluded that it seems that fennel has estrogen like potential.

## 3. Challenges and Way Forward

Due to the complex composition, conducting systematic and thorough research evaluating the efficacy and safety of phytogenics is still difficult [119]. Furthermore, consistencies in the obtained results can be attributed to a variety of factors, including the source and bioactive compounds, which can vary depending on the plant, origin, growing areas, preparation methods, storage conditions, the absorbed dose, climatic conditions, management, and experimental design [120].

Furthermore, due to their volatile and reactive nature, the essential oils may evaporate quickly, resulting in a misguided concentration in the final feed additive. As a result, preserving their stability, as well as maintaining their biological activity, presents a very difficult task. Furthermore, reciprocal interactions with other feed matrix substances have been identified, such as reduced biological effects of PFA in fibrous or high protein diets [121]. Several phytogenic chemicals have also been demonstrated to be substantially absorbed in the upper GIT, implying that without adequate protection, the majority would not reach the lower gut, where they would perform their primary roles. Novel delivery technologies such as microencapsulation have received much attention as a novel delivery technology, which protects the phytogenics from oxidation and degradation.

The published literature on fennel seeds showed variable results in terms of growth performance and health effects. Interestingly, in most instances, fennels seeds preparations have been used singly, while the recent research findings indicate that synergistic interaction is of great importance to maximize the efficiency of the combined doses in the best possible manner. To assess their potential for application in poultry production, scientists must first determine the specific mechanism of action of fennel at the molecular level. To begin with, phytogenic chemical bioavailability is still a contentious issue. The metabolism of phytogenics in birds is similarly a poorly understood subject. Furthermore, phytogenic metabolism produces a huge variety of chemicals with different chemical structures, making it difficult to determine their specific effects and mechanisms of action. Another potential issue is that most products on the market are multi-ingredient, making it difficult to evaluate the effects of utilizing specific components and distinguishing between them. Another issue is determining the best amounts for poultry, especially because most chemicals are included in feed or water, making it impossible to monitor individual bird consumption. As a low dosage may not be beneficial, while a large amount may already be harmful, the doses are critical for achieving the intended result. Another factor to consider is the possibility of interactions between phytogenic and other feed additives. Phytogenic component stability after feed processing is also frequently questioned. Another point worth emphasizing is that, while there are countless examples of beneficial supplementation with phytogenic preparations, there are also a few studies that show that this sort of dietary therapy has no impact. There is very little information known on the chemicals’ safety and residual toxicity.

Several studies have shown encouraging results, but more attention needs to be paid to the identification of active chemicals in order to create potentially successful blends. Furthermore, the most significant variables guiding efficiency by managing both the time and location of the release of active chemicals include selecting the suitable protective approach.

## 4. Conclusions

From the current review, it was concluded that fennels seeds supplementation has multiple beneficial impacts on poultry growth and health. There are, however, some limits that must be recognized. The difficulties of bioavailability, plant derivative metabolism in birds and the difficulty of standardizing commercial products are the most important concerns. Numerous studies have previously identified positive effects of fennel seeds preparation on the health of chickens, and practical application in poultry production. Supplementing chicken feed with fennel seeds has been found to boost poultry health and productivity while also protecting them from several infectious diseases. However, additional studies into exact dose, bioavailability of effective compounds, and novel delivery technologies are the futuristic focus of scientists.

## Figures and Tables

**Table 1:** Classification and description of fennel plant (https://plants.usda.gov accessed 11 February 2022).

**Kingdom**	***Plantae***	**Plant family**	*Apiacea*	**Sowing time**	March April
**Division**	*Magnoliophyta*	**Plant height approx.**	40–200 cm	**Best germination temperature**	15–20 °C
**Class**	*Magnoliopsida*	**Flowering time**	July August September	**Germination time in days**	7–14
**Order**	*Apiales*	**Flower color**	Yellow	**Planting distance**	20–60 cm
**Family**	*Apiaceae (Umbelliferae)*	**Root system**	Taproot	**Bad intercropping partner**	Dill
**Genus**	*Foeniculum*	**Lifecycle**	Biennial Perennial Vivacious		
**Species**	*Foeniculum vulgare*	**Sunlight**	Full sun		

**Table 2:** Proximate composition and important bioactive constituents in fennel seeds [52,53].

Nutrient Composition	Quantity/100 g	Minerals	Concentration, mg	Vitamins	Concentration
Moisture	90.21	Calcium, Ca	49	Vitamin C	12 mg
Energy	31 kcal	Iron, Fe	0.73	Thiamin B-1	0.01 mg
Protein	1.24	Magnesium, Mg	17	Riboflavin B-2	0.032 mg
Total lipid (fat)	0.2	Phosphorus, P	50	Niacin B-3	0.64 mg
Carbohydrate	7.3	Potassium, K	414	Vitamin B-6	0.047 mg
Total dietary fiber	3.1	Sodium, Na	52	Folate	27 µg
Sugars	3.93	Zinc, Zn	0.2	Vitamin A	48 µg
**Lipids**				Vitamin E	0.58 mg
Fatty acids, total saturated	0.09	**Essential amino acids**	**Concentration, mg**	**Nonessential amino acid**	**Concentration, mg**
Fatty acid, total monounsaturated	0.068	Leucine	0.63	Glycine	0.55
Fatty acids, total polyunsaturated	0.169	Isoleucine	0.73	Proline	0.53
		Phenylalanine	0.45		
		Tryptophane	0.53		
**Essential oils (% of total oil)**					
**Monoterpenes**		**Oxygenated monoterpene**			
α-thujene	0.14	1,8-cineol	0.17		
α-pinene	0.37	Fenchone	10.99		
Camphene	0.08	Linalool	0.11		
Sabinene	0.14	Fenchyl alcohol	0.04		
β-pinene	0.05	α–thujone	0.04		
β-myrcene	0.81	Camphor	0.47		
α-phellandrene	0.18	Estragole	7.17		

**Table 3:** Reported beneficial effects of fennel in poultry.

Parameters	Dose	Source	Poultry Species	Effect	Reference
**Feed Intake**	1.2 and 3.2%	Fennel seed pow-der	Broilers	Increased	Al-Sagon et al. [61]
	0.15 and 0.25 g/kg	Fennel essential oil	Broilers	Increased	Gharehsheikhlou et al. [62]
	1 and 2%	Fennel seed	Broiler	Increased	Ragab [63]
	40 mg/kg	Fennel extract	Laying hens	Increased	Vakili [64]
	0.25 and 0.5%	Fennel seed Pow-der	Broilers	Increased	Saki et al. [65]
	0.25, 0.50 and 0.75 g/kg	Fennel seed meal	Japanese quail	Increased	Henda et al. [66]
	1.0%	Fennel seed	Japanese quail	Increased	Ragab [67]
	5 g/kg	Fennel seed	Laying hens	Decreased	Abou-Elkhair et al. [68]
	2.5%	Fennel seed pow-der	Broilers	Decreased	Zahira Abul-Jabbar et al. [69]
	10 and 20 g/kg	Fennel seed fruit	Laying hens	No effect	Gharghani et al. [70]
	0.25 to 1.5 g/kg	Fennel seed	Broilers	No effect	Soltan et al. [71]
	200 mg/kg	Fennel essential oil	Broilers	Increased	A. R. Ghiasvand et al. [72]
	5–10%	Fennel seed	Broilers	Increased	Milica et al. [73]
	0.3, 0.6 and 0.9%	Fennel seeds	Broiler	No effect	Bugdaycı et al. [74]
	24 mg/kg	Essential oil	Laying hens	No effect	Cabuk et al. [75]
	250 to 750 g/50 kg	Fennel seed	Broilers	Increased	Saleh Lamarb et al. [76]
**Feed Efficiency**	24 mg/kg	Fennel essential oil	Broilers	No effect	Cabuk et al. [77]
	1.2 and 3.2%	Fennel seed pow-der	Broilers	Improved	Al-Sagan et al. [61]
	100 mg/kg	Fennel essential oil	Broilers	Improved	Cengiz et al. [78]
	5%	Fennel seed pow-der	Broilers	Improved	Zahira Abul-Jabbar et al. [69]
	0.25 and 0.5%	Fennel seed pow-der	Broilers	Improved	Saki et al. [65]
	1, 2 and 3 g/kg	Fennel seed	Broilers	Improved	Abdullah and Abbas [79]
	5%, 10% or 15%	MOL	Japanese quail	Improved	Ragab [67]
	300 mg	Fennel essential oil	Laying hens	No effect	Nasiroleslami et al. [80]
	0.3, 0.6 and 0.9%	Fennel seed	Laying quails	No effect	Bugdaycı et al. [74]
	5 g/kg	Fennel seed	Laying hens	Improved	Abou-Elkhair et al. [68]
	0.25 and 0.5%	Fennel seed pow-der	Broilers	Improved	Saki et al. [65]
	1, 2 and 3 g/kg	Fennel seed	Broilers	No effect	Abdullah and Abbas [79]
	100 to 400 ppm	Fennel extract	Broilers	Not effected	Ali Safaei et al. [81]
	250 to 750 g/50 kg	Fennel seed	Broilers	Improved	Saleh Lamarb et al. [76]
**Body Weight**	24 mg/kg	Essential oil	Laying hens	Improved	Cabuk et al. [77]
	0.3, 0.6 and 0.9%	Fennel seed	Laying quails	No effect	Bugdaycı et al. [74]
	1, 2 and 3 g/kg	Fennel seed	Broilers	Increased	Abdullah and Abbas [79]
	1%	Fennel seed with kemzyme dry and CP	Japanese quails	Increased	Ragab [67]
	10 and 20 g/kg	Fennel fruit	Laying hens	Increased	Gharaghani et al. [70]
	0.25, 0.5 and 0.75 g/kg	Fennel seed meal	Japanese quails	Increased	Henda et al. [66]
	0.5, 1.0, and 1.5%	Fennel seed pow-der	Japanese quails	Increased	Premavalli et al. [81]
	5 g/kg	Fennel seed	Laying hens	No effect	Abou-Al-khair et al. [68]
	100 to 400 ppm	Fennel extract	Broilers	Increased	Ali Safaei et al. [81]
**Growth Performance**	250 to 750 g/50 kg	Fennel seed	Broilers	Increased	Saleh Lamarb et al. [76]
	1.2 and 3.2%	Fennel seed pow-der	Broilers	Increased	Al-Sagon et al. [61]
	1%	Fennel seed	Japanese quails	Increased	Ragab [67]
	0.15 and 0.25 g/kg	Fennel essential oil	Broilers	Increased	Gharehsheikhlou et al. [62]
	300 mg/kg	Fennel essential oil	Laying hens	No effect	Nasiroleslami et al. [80]
	250, 500 and 750 g/50 kg	Fennel seed	Broilers	Improved	Saleh Lamarb et al. [76]
**Carcass Traits/Dressing Percentage**	10 and 20 g/kg	Fennel fruit	Laying hens	Increased	Gharghani et al. [70]
	100 mg/kg	Fennel essential oil	Broilers	Improved	Cengis et al. [78]
	1.2 and 3.2%	Fennel seed pow-der	Broilers	Improved	Al-Sagan et al. [61]
	1, 2 and 3 g/kg	Fennel seed	Broilers	No effect except Pancreas and stomach weight percentage	Abdullah and Abbas [79]
	0.5 and 1%	Fennel seed	Japanese quails	Improved	Ragab et al. [67]
	0.25, 0.50 and 0.75 g/kg	Fennel Seed Meal	Japanese quails	Improved	Henda et al. [66]
	100 mg/kg	Fennel oil	Broilers	No effect	Cengis et al. [78]
	200 mg/kg	Fennel essential oil	Broilers	No effect	A. R. Ghiasvand et al. [72]
**Egg Production and Quality**	0.15 and 0.25 g/kg	Fennel essential oil	Broilers	Improved	Gharehsheikhlou et al. [62]
	50 mg/kg	Fennel Extract	Broiler breeder	Improved	Kazemi et al. [82]
	10 mg/kg	Fennel seed ex-tract	Laying hens	Improved	Raza et al. [83]
	300 mg/kg	Fennel essential oil	Laying hens	No effect on egg index and yolk index, improved egg shell weight and thickness Haugh unit decreased	Nasiroleslami et al. [80]
	24 mg/kg	Fennel essential oil	Laying hens	Improved	Cabuk et al. [75]
	0.3, 0.6 and 0.9%	Fennel seed	Laying quails	No effect	Bugdaycı et al. [74]
**Immunity**	24 mg/kg	Fennel essential oil	Laying hens	Improved	Cabuk et al. [77]
	50 mg/kg	Fennel extract	Broiler breeder	Improved	Kazemi et al. [84]
	36 mg/kg	Fennel seed	Laying hens	Improved	K-Ozek [85]
		Fennel essential oil	Broilers		Ghiasvand et al. [72]
	48 mg/kg	Essential oil	Broilers	No effect on antibody titer against IBD and ND	Bozkurt et al. [86]
**Relative Weight of Lymphoid Organs**	100, 200, 300 and 400 ppm	Fennel extract	Broilers	ND, IBD titer improved	Ali Safaei et al. [81]
	60–120 ml/liter	Fennel seed meal	Japanese quails	Improved	Henda et al. [66]
	0.5 and 1%	Fennel seed	Japanese quails	Improved	Ragab [67]
	0.3 ml of fennel oil/kg	Fennel essential oil	Broilers	Improved	Zahira Abul-Jabbar et al. [69]
	48 mg/kg	Essential oil	Broilers	No effect on relative weight of liver and Bursa	Bozkurt et al. [86]
	1 and 2%	Fennel seed	Broilers	Improved	Ragab [63]
	10 mg/kg	Fennel seed ex-tract	Laying hens	Improved	Raza et al. [83]
	1, 2 and 3 g/kg	Fennel seed	Broilers	Improved	Abdullah and Abbas [79]
**Antioxidant Activity**	1.2 and 3.2%	Fennel seed pow-der	Broilers	Decreased MDA concentration	Al-Sagan et al. [61]
	5 g/kg	Fennel seed	Laying hens	Decreased MDA concentration	Abou-Al-Khair et al. [68]
	10 and 20 g/kg	Fennel fruit	Laying hens	Decreased MDA concentration	Gharaghani et al. [70]
**Blood Biochemistry**	1%	Ground Fennel seed	Broilers	Decreased MDA concentration	Gharaghani et al. [87]
	1, 2 and 3 g/kg	Fennel seed	Broilers	Higher RBC count, Hb and PCV	Abdullah and Abbas [79]
	0.5 and 1%	Fennel seed	Japanese quails	Higher contents of serum glucose, tri-glycerides, aspartate aminotransferase, alanine aminotransferase, total protein and albumin	Ragab.S et al [67]
	1 and 2%	Fennel seed	Broilers	Improved leukocyte count	Ragab [63]
	5%	Fennel seed pow-der	Broilers	Lower concentration of glucose, tri-glycerides and uric acid	Zahira Abul-Jabbar et al. [69]
	10 mg/kg	Fennel seed ex-tract	Laying hens	No effect on cholesterol and triglyceride	Raza et al. [83]
	100, 200, 300 and 400 ppm	Fennel extract	Broilers	No effect on concentration of glucose, triglyceride, LDL and alkaline phos-phatase while HDL increased, and uric acid decreased	Ali Safaei et al. [81]
	0.25, 0.5 and 0.75 g/kg	Fennel seed meal	Japanese quails	Non-significant increase in serum total protein albumin and globulin	Henda et al. [66]
**Economics Efficiency**	0.15 and 0.25 g/kg	Fennel essential oil	Broilers	Improved the total cholesterol/HDL ratio and LDL/HDL ratio	Gharehsheikhlou et al. [62]
	200 mg/kg	Fennel essential oil	Broilers	No effect on blood lymphocyte and heterophil percentages and heterophil to lymphocyte ratio	A. R. Ghiasvand et al. [72]
	3.2%	Fennel seed pow-der	Broilers	Increased net profit	Al-Sagon et al. [61]
	0.25, 0.5 and 0.75 g/kg	Fennel seed meal	Japanese quails	Improved	Henda et al. [66]
